# Supplementary material for: Efficacy and safety of single-dose 40 mg/kg oral praziquantel in the treatment of schistosomiasis in preschool-age versus school-age children: An individual participant data meta-analysis
Source: PLoS Negl Trop Dis. 2020 Jun 22;14(6):e0008277. doi: 10.1371/journal.pntd.0008277 (PMC7360067; doi:10.1371/journal.pntd.0008277)
Supplement: S1 Table — (DOCX) [file pntd.0008277.s001.docx]

S1 Table. Species and diagnostic approach used (excluded studies shaded)

| Author, Year of study [REF] | Country | Species | Diagnostic approach |
| --- | --- | --- | --- |
| Coulibaly, 2011 | CÔTE D’IVOIRE | *S. mansoni S. haematobium* | duplicate 41.7 mg Kato-Katz thick smears from 2 stools single filtration from 2 urine samples |
| Coulibaly, 2017 | CÔTE D’IVOIRE | S. mansoni | duplicate 41.7 mg Kato-Katz thick smears from 2 stools |
| Coulibaly, 2018 | CÔTE D’IVOIRE | S. haematobium | single filtration from 3 urine samples |
| Garba, 2007 | NIGER | *S. mansoni S. haematobium* | single 41.7 mg Kato-Katz thick smears from 3 stools single filtration from 3 urine samples |
| Garba, 2009 | NIGER | *S. haematobium* | single filtration from 3 urine samples |
| Garba, 2013 | NIGER | *S. mansoni S. haematobium* | single 41.7 mg Kato-Katz thick smears from 3 stools single filtration from 3 urine samples |
| Goran, 2000 | CÔTE D’IVOIRE | *S. haematobium* | single filtration from 4 urine samples |
| Landouré, 2006 | MALI | *S. mansoni S. haematobium* | duplicate 41.7 mg Kato-Katz thick smears from 1 stool single filtration from 2 urine samples for 2 days |
| Landouré, 2009 | MALI | *S. mansoni S. haematobium* | duplicate 41.7 mg Kato-Katz thick smears from 1 stool single filtration from 2 urine samples |
| Lossa, 1996 | NIGER | *S. haematobium* | single filtration from 2 urine samples |
| Mutapi, 2010 | ZIMBABWE | *S. mansoni S. haematobium* | single 41.7 mg Kato-Katz thick smears from 3 stools single filtration from 3 urine samples |
| Niame, 1995 | NIGER | *S. mansoni S. haematobium* | single 41.7 mg Kato-Katz thick smears from 1 stool single filtration from 1 urine sample |
| Olds, 1990 | KENYA | *S. haematobium* | duplicate filtrations from 2 daily urines |
| Olliaro, 2007 | BRAZIL  PHILIPPINES  MAURITANIA | *S. mansoni S. japonicum* | duplicate 41.7 mg Kato-Katz thick smears from 2 stools single filtration from 2 urine samples |
| Raso, 2004 | MALI | *S. mansoni* | duplicate 41.7 mg Kato-Katz thick smears from 3 stools |
| Sacko, 2009 | MALI | *S. mansoni S. haematobium* | duplicate 41.7 mg Kato-Katz thick smears from 2 stools single filtration from 2 urine samples |
| Scherrer, 2007 | CÔTE D’IVOIRE | *S. mansoni* | duplicate 41.7 mg Kato-Katz thick smears |
| Sousa-Figueiredo, 2012 | UGANDA | *S. mansoni* | duplicate 41.7 mg Kato-Katz thick smears from 2 stools |
| Stete, 2010 | CÔTE D’IVOIRE | *S. haematobium* | single filtration from 3 urine samples |
| Utzinger, 1997 | CÔTE D’IVOIRE | *S. mansoni* | single 41.7 mg Kato-Katz thick smears from 4 stools |
| Utzinger, 1998 | CÔTE D’IVOIRE | *S. mansoni* | single 41.7 mg Kato-Katz thick smears from 4 stools |
| Wami, 2014 | ZIMBABWE | *S. mansoni S. haematobium* | single 41.7 mg Kato-Katz thick smears from 2 stools single filtration from 3 urine samples |
| Zhou, 2007 | CHINA | *S. japonicum* | Serological positives were tested by Kato-Katz technique |
