# Supplementary material for: Efficacy and safety of single-dose 40 mg/kg oral praziquantel in the treatment of schistosomiasis in preschool-age versus school-age children: An individual participant data meta-analysis
Source: PLoS Negl Trop Dis. 2020 Jun 22;14(6):e0008277. doi: 10.1371/journal.pntd.0008277 (PMC7360067; doi:10.1371/journal.pntd.0008277)
Supplement: S2 Table — (DOCX) [file pntd.0008277.s002.docx]

S2 table. Intensity of infection and treatment outcomes by study for *Schistosoma mansoni*

| **Reference** | **Age category** | **N evaluable** | **Mean EPG at baseline** | **Mean EPG at follow-up** | **Egg reduction rate ERR 95%CI** | **Cure rate CR 95%CI** | **Mean individual egg reduction rate ERR 95%CI** |
| --- | --- | --- | --- | --- | --- | --- | --- |
| Coulibaly 2011 | [0-6[ | 35 | 4.64 | 0.03 | 99.38 ( 98.40; 100.00) | 91.43% ( 82.15% ; 100.0% ) | 97.76 ( 94.56; 100.97) |
| Coulibaly 2017 | [0-6[ | 38 | 2.71 | 0.36 | 86.89 ( 67.82; 96.93) | 73.68% ( 59.68% ; 87.68% ) | 85.00 ( 74.42; 95.58) |
| Coulibaly 2017 | [6-10[ | 22 | 7.95 | 0.26 | 96.71 ( 92.86; 99.30) | 68.18% ( 48.72% ; 87.64% ) | 95.08 ( 91.08; 99.08) |
| Coulibaly 2017 | [10-14] | 20 | 10.93 | 0.4 | 96.34 ( 89.66; 98.80) | 65.00% ( 44.10% ; 85.90% ) | 83.00 ( 66.08; 99.92) |
| Garba 2007 | [6-10[ | 99 | 139.19 | 36.32 | 73.90 ( 65.50; 81.44) | 54.55% ( 44.74% ; 64.35% ) | 74.58 ( 67.72; 81.43) |
| Garba 2007 | [10-14] | 82 | 218.73 | 28.73 | 86.86 ( 79.81; 93.09) | 65.85% ( 55.59% ; 76.12% ) | 84.93 ( 78.58; 91.28) |
| Garba 2013 | [0-6[ | 88 | 114.59 | 7.82 | 93.18 ( 88.93; 96.20) | 75.00% ( 65.95% ; 84.05% ) | 86.16 ( 79.44; 92.87) |
| Olliaro 2007 | [10-14] | 190 | 25.14 | 0.79 | 96.88 ( 93.45; 99.32) | 90.53% ( 86.36% ; 94.69% ) | 98.09 ( 96.56; 99.61) |
| Raso 2004 | [0-6[ | 4 | 0.58 | 0 | 100.00 (100.00; 100.00) | 100.0% ( 100.0% ; 100.0% ) | 100.00 ( . ; . ) |
| Raso 2004 | [6-10[ | 12 | 3.5 | 0.08 | 97.62 ( 83.05; 100.00) | 75.00% ( 50.50% ; 99.50% ) | 89.96 ( 71.75; 108.16) |
| Raso 2004 | [10-14] | 22 | 4.5 | 1.47 | 67.34 ( 26.10; 90.82) | 54.55% ( 33.74% ; 75.35% ) | 76.08 ( 59.21; 92.95) |
| Scherrer 2007 | [0-6[ | 6 | 3.33 | 0.08 | 97.50 ( 60.00; 100.00) | 83.33% ( 53.51% ; 100.0% ) | 83.33 ( 40.49; 126.18) |
| Scherrer 2007 | [6-10[ | 22 | 7.32 | 0.05 | 99.38 ( 96.40; 100.00) | 90.91% ( 78.90% ; 100.0% ) | 94.76 ( 85.26; 104.25) |
| Scherrer 2007 | [10-14] | 21 | 2.24 | 1.33 | 40.43 (-95.09; 100.00) | 85.71% ( 70.75% ; 100.0% ) | 88.62 ( 74.66; 102.57) |
| Sousa-Figueiredo 2012 | [0-6[ | 211 | 334.48 | 70.27 | 78.99 ( 67.43; 87.87) | 51.18% ( 44.44% ; 57.93% ) | 80.00 ( 75.38; 84.63) |
| Sousa-Figueiredo 2012 | [6-10[ | 94 | 328.79 | 34.79 | 89.42 ( 76.96; 95.99) | 68.09% ( 58.66% ; 77.51% ) | 86.91 ( 81.01; 92.81) |
| Utzinger 1997 | [6-10[ | 27 | 2.25 | 0.07 | 96.98 ( 93.24; 98.99) | 70.37% ( 53.15% ; 87.59% ) | 89.99 ( 80.85; 99.13) |
| Utzinger 1997 | [10-14] | 56 | 4.65 | 0.2 | 95.78 ( 92.13; 98.48) | 71.43% ( 59.60% ; 83.26% ) | 95.03 ( 89.98; 100.07) |
