# Supplementary material for: Efficacy and safety of single-dose 40 mg/kg oral praziquantel in the treatment of schistosomiasis in preschool-age versus school-age children: An individual participant data meta-analysis
Source: PLoS Negl Trop Dis. 2020 Jun 22;14(6):e0008277. doi: 10.1371/journal.pntd.0008277 (PMC7360067; doi:10.1371/journal.pntd.0008277)
Supplement: S3 Table — (DOCX) [file pntd.0008277.s003.docx]

S3 table. Intensity of infection and treatment outcomes by study for *Schistosoma haematobium*

| **Reference** | **Age category** | **N evaluable** | **Mean EPG at baseline** | **Mean EPG at follow-up** | **Egg reduction rate ERR 95%CI** | **Cure rate CR 95%CI** | **Mean individual egg reduction rate ERR 95%CI** |
| --- | --- | --- | --- | --- | --- | --- | --- |
| Coulibaly 2011 | [0-6[ | 18 | 13.44 | 0.08 | 99.38 (99.00; 100.00) | 94.44% (83.86% ; 100.0%) | 99.94 (99.80; 100.07) |
| Coulibaly 2018 | [0-6[ | 37 | 23.42 | 0.44 | 98.12 (95.76; 99.56) | 75.68% (61.85% ; 89.50%) | 92.25 (84.22; 100.28) |
| Coulibaly 2018 | [6-10[ | 25 | 35.51 | 0.23 | 99.36 (98.77; 99.78) | 68.00% (49.71% ; 86.29%) | 99.33 (98.85; 99.82) |
| Coulibaly 2018 | [10-14] | 12 | 25.76 | 0.29 | 98.87 (98.06; 100.00) | 75.00% (50.50% ; 99.50%) | 99.26 (98.28; 100.23) |
| Garba 2007 | [6-10[ | 211 | 34.73 | 1.22 | 96.49 (94.31; 97.94) | 69.19% (62.96% ; 75.42%) | 96.25 (94.62; 97.87) |
| Garba 2007 | [10-14] | 157 | 27.49 | 1.06 | 96.16 (94.12; 97.81) | 69.43% (62.22% ; 76.63%) | 96.73 (95.54; 97.92) |
| Garba 2009 | [6-10[ | 177 | 105.74 | 2.25 | 97.87 (96.88; 98.62) | 51.98% (44.62% ; 59.34%) | 94.57 (92.25; 96.89) |
| Garba 2009 | [10-14] | 170 | 103.97 | 7 | 93.27 (84.59; 98.13) | 48.82% (41.31% ; 56.34%) | 91.61 (88.27; 94.94) |
| Garba 2013 | [0-6[ | 161 | 32.74 | 0.31 | 99.05 (98.18; 99.51) | 85.71% (80.31% ; 91.12%) | 96.39 (94.02; 98.76) |
| Lossa 1996 | [0-6[ | 61 | 14.89 | 22.39 | -50.36 (-148.6; 36.71) | 54.10% (41.59% ; 66.60%) | 75.74 (65.51; 85.96) |
| Lossa 1996 | [6-10[ | 85 | 79.06 | 66.45 | 15.94 (-35.32; 52.69) | 22.35% (13.50% ; 31.21%) | 59.58 (49.98; 69.18) |
| Lossa 1996 | [10-14] | 75 | 81.42 | 35.05 | 56.96 (34.95; 72.27) | 25.33% (15.49% ; 35.18%) | 61.54 (52.19; 70.90) |
| Mutapi 2010 | [0-6[ | 21 | 69.73 | 42.56 | 38.96 (-8.31; 97.07) | 76.19% (57.97% ; 94.41%) | 77.58 (58.76; 96.40) |
| Mutapi 2010 | [6-10[ | 115 | 75.54 | 5.16 | 93.17 (85.67; 97.47) | 84.35% (77.71% ; 90.99%) | 93.45 (89.26; 97.64) |
| Mutapi 2010 | [10-14] | 34 | 60.52 | 11.9 | 80.34 (22.66; 99.59) | 79.41% (65.82% ; 93.00%) | 91.69 (82.67; 100.72) |
| Niame 1995 | [6-10[ | 30 | 108.5 | 0 | 100.00 (100.00; 100.00) | 100.0% (100.0% ; 100.0%) | 100.00 ( . ; .) |
| Niame 1995 | [10-14] | 60 | 50.33 | 0 | 100.00 (100.00; 100.00) | 100.0% (100.0% ; 100.0%) | 100.00 ( . ; .) |
| Olds, 1999 | [0-6[ | 2 | 33 | 6.5 | 80.30 (30.00; 89.29) | 0.000% (0.000% ; 0.000%) | 59.64 (-317.0; 436.29) |
| Olds, 1999 | [6-10[ | 49 | 240.86 | 122.84 | 49.00 (24.26; 71.85) | 38.78% (25.13% ; 52.42%) | 71.14 (59.38; 82.89) |
| Olds, 1999 | [10-14] | 67 | 275.06 | 106.8 | 61.17 (38.34; 77.67) | 29.85% (18.89% ; 40.81%) | 70.66 (61.21; 80.10) |
| Stete 2010 | [0-6[ | 1 | 9.5 | 0 | 100.00 (100.00; 100.00) | 100.0% (100.0% ; 100.0%) | 100.00 ( . ; .) |
| Stete 2010 | [6-10[ | 20 | 74.4 | 0.25 | 99.66 (99.27; 100.00) | 90.00% (76.85% ; 100.0%) | 99.91 (99.77; 100.04) |
| Stete 2010 | [10-14] | 56 | 91.96 | 2.66 | 97.10 (94.24; 99.28) | 76.79% (65.73% ; 87.84%) | 97.39 (94.67; 100.10) |
