# Supplementary material for: Efficacy and safety of single-dose 40 mg/kg oral praziquantel in the treatment of schistosomiasis in preschool-age versus school-age children: An individual participant data meta-analysis
Source: PLoS Negl Trop Dis. 2020 Jun 22;14(6):e0008277. doi: 10.1371/journal.pntd.0008277 (PMC7360067; doi:10.1371/journal.pntd.0008277)
Supplement: S4 Table — (DOCX) [file pntd.0008277.s004.docx]

S4 table. General linear model of individual ERR with baseline log-transformed egg count as covariate and studies, age and sex as factors

|  |  | ***S. mansoni*** | | |  | ***S. haematobium*** | | |
| --- | --- | --- | --- | --- | --- | --- | --- | --- |
| **Effect** | **Category** | **Mean** | **SD** | **Pr > \|t\|** | **Category** | **Mean** | **SD** | **Pr > \|t\|** |
| **Intercept** |  | 0.9562 | 0.04815 | <.0001 |  | 0.9978 | 0.05670 | <.0001 |
| **Ln(Baseline EPG)** |  | 0.02950 | 0.007335 | <.0001 |  | 0.004921 | 0.003800 | 0.1954 |
| **Study** | **Coulibaly 2017** | -0.1360 | 0.05787 | 0.0190 | **Coulibaly 2018** | -0.03142 | 0.06390 | 0.6230 |
|  | **Garba 2007** | -0.3414 | 0.06326 | <.0001 | **Garba 2007** | -0.00941 | 0.06305 | 0.8814 |
|  | **Garba 2013** | -0.2086 | 0.05956 | 0.0005 | **Garba 2009** | -0.04544 | 0.06327 | 0.4727 |
|  | **Olliaro 2007** | -0.1037 | 0.06320 | 0.1012 | **Garba 2013** | -0.03584 | 0.05915 | 0.5447 |
|  | **Raso 2004** | -0.1866 | 0.07044 | 0.0082 | **Olds, 1999** | -0.2736 | 0.06598 | <.0001 |
|  | **Scherrer 2007** | -0.1055 | 0.06638 | 0.1122 | **Lossa 1996** | -0.3335 | 0.06130 | <.0001 |
|  | **Sousa-Figueiredo 2012** | -0.2707 | 0.05580 | <.0001 | **Mutapi 2010** | -0.06966 | 0.06332 | 0.2715 |
|  | **Utzinger 1997** | -0.08750 | 0.06410 | 0.1726 | **Niame 1995** | 0.02945 | 0.06688 | 0.6597 |
|  |  |  |  |  | **Stete 2010** | 0.006912 | 0.06764 | 0.9186 |
|  | **Coulibaly 2011** | 0.0000 | . | . | **Coulibaly 2011** | 0.0000 | . | . |
| **Age** | **[10-14]** | 0.05331 | 0.03718 | 0.1520 | **[10-14]** | -0.03681 | 0.02728 | 0.1774 |
|  | **[6-10[** | 0.04144 | 0.02883 | 0.1508 | **[6-10[** | -0.02624 | 0.02656 | 0.3234 |
|  | **[0-6[** | 0.0000 | . | . | **[0-6[** | 0.0000 | . | . |
| **Sex** | **Male** | -0.02341 | 0.01727 | 0.1757 | **Male** | -0.01338 | 0.01188 | 0.2603 |
|  | **Female** | 0.0000 | . | . | **Female** | 0.0000 | . | . |
