# Supplementary material for: Efficacy and safety of single-dose 40 mg/kg oral praziquantel in the treatment of schistosomiasis in preschool-age versus school-age children: An individual participant data meta-analysis
Source: PLoS Negl Trop Dis. 2020 Jun 22;14(6):e0008277. doi: 10.1371/journal.pntd.0008277 (PMC7360067; doi:10.1371/journal.pntd.0008277)
Supplement: S5 Table — (DOCX) [file pntd.0008277.s005.docx]

S5 table. Mixed model of individual *S. mansoni* and *S. haematobium* baseline log-transformed egg count with random effect for the study

|  |  | ***S. mansoni*** | | |  | ***S. haematobium*** | | |
| --- | --- | --- | --- | --- | --- | --- | --- | --- |
| **Effect** | **Category** | **Mean** | **SD** | **Pr > \|t\|** | **Category** | **Mean** | **SD** | **Pr > \|t\|** |
| **Intercept** |  | 2.1838 | 0.5912 | 0.0061 |  | 2.3424 | 0.2263 | <.0001 |
| **Age** | **[10-14]** | 0.5619 | 0.1559 | 0.0003 | **[10-14]** | 0.5857 | 0.1686 | 0.0005 |
|  | **[6-10[** | 0.2956 | 0.1215 | 0.0152 | **[6-10[** | 0.6313 | 0.1644 | 0.0001 |
|  | **[0-6[** | 0 | . | . | **[0-6[** | 0 | . | . |
| **SEX** | **Male** | -0.01079 | 0.07323 | 0.8829 | **Male** | 0.1936 | 0.07725 | 0.0123 |
|  | **Female** | 0 | . | . | **Female** | 0 | . | . |
| **Intercept** |  | 2.2593 | 0.5811 | 0.005 |  | 2.3385 | 0.2253 | <.0001 |
| **Age** | **[6-14]** | 0.3373 | 0.1203 | 0.005 | **[6-14]** | 0.6136 | 0.1604 | 1E-04 |
|  | **[0-6[** | 0 | . | . | **[0-6[** | 0 | . | . |
| **SEX** | **Male** | -0.01286 | 0.07338 | 0.861 | **Male** | 0.194 | 0.07723 | 0.012 |
|  | **Female** | 0 | . | . | **Female** | 0 | . | . |
