# Supplementary material for: Efficacy and safety of single-dose 40 mg/kg oral praziquantel in the treatment of schistosomiasis in preschool-age versus school-age children: An individual participant data meta-analysis
Source: PLoS Negl Trop Dis. 2020 Jun 22;14(6):e0008277. doi: 10.1371/journal.pntd.0008277 (PMC7360067; doi:10.1371/journal.pntd.0008277)
Supplement: S6 Table — Results of general linear model of individual a) S. mansoni and b) S. haematobium baseline log transformed egg count with study, age, and sex as factors. (DOCX) [file pntd.0008277.s006.docx]

S6 table. General linear model of individual *S. mansoni* and *S. haematobium* baseline log-transformed egg count with study, age, and sex as factors(a: 3 categories, b: 2 categories)

| a |  | ***S. mansoni*** | | |  | ***S. haematobium*** | | |
| --- | --- | --- | --- | --- | --- | --- | --- | --- |
| **Effect** | **Category** | **Mean** | **SD** | **Pr > \|t\|** | **Category** | **Mean** | **SD** | **Pr > \|t\|** |
| **Intercept** |  | 1.1616 | 0.2009 | <.0001 |  | 1.5792 | 0.3674 | <.0001 |
| **Study** | **Coulibaly 2017** | -0.06718 | 0.2453 | 0.7843 | **Coulibaly 2018** | 0.6585 | 0.4161 | 0.1138 |
|  | **Garba 2007** | 3.174 | 0.2494 | <.0001 | **Garba 2007** | 0.3295 | 0.4108 | 0.4226 |
|  | **Garba 2013** | 3.1392 | 0.2329 | <.0001 | **Garba 2009** | 1.1824 | 0.4113 | 0.0041 |
|  | **Olliaro 2007** | 1.1118 | 0.2657 | <.0001 | **Garba 2013** | 0.3262 | 0.3854 | 0.3975 |
|  | **Raso 2004** | -0.5553 | 0.2981 | 0.0628 | **Olds, 1999** | 2.2444 | 0.4264 | <.0001 |
|  | **Scherrer 2007** | -0.5092 | 0.281 | 0.0702 | **Lossa 1996** | 0.5077 | 0.3993 | 0.2037 |
|  | **Sousa-Figueiredo 2012** | 3.4087 | 0.2115 | <.0001 | **Mutapi 2010** | 0.9035 | 0.4121 | 0.0285 |
|  | **Utzinger 1997** | -0.6146 | 0.2711 | 0.0236 | **Niame 1995** | 0.8256 | 0.4353 | 0.0581 |
|  | **Coulibaly 2011** | 0 |  |  | **Stete 2010** | 1.1619 | 0.4399 | 0.0083 |
|  |  |  | . | . | **Coulibaly 2011** | 0 | . | . |
| **Age** | **[10-14]** | 0.5767 | 0.1566 | 0.0002 | **[6-14]** | 0.4883 | 0.1773 | 0.006 |
|  | **[6-10[** | 0.3034 | 0.1219 | 0.0129 | **[6-10[** | 0.5412 | 0.1726 | 0.0017 |
|  | **[0-6[** | 0 | . | . | **[0-6[** | 0 | . | . |
| **Sex** | **Male** | -0.00971 | 0.07324 | 0.8946 | **Male** | 0.1932 | 0.07728 | 0.0125 |
|  | **Female** | 0 | . | . | **Female** | 0 | . | . |

| b |  | ***S. mansoni*** | | |  | ***S. haematobium*** | | |
| --- | --- | --- | --- | --- | --- | --- | --- | --- |
| **Effect** | **Category** | **Mean** | **SD** | **Pr > \|t\|** | **Category** | **Mean** | **SD** | **Pr > \|t\|** |
| **Intercept** |  | 1.1627 | 0.2014 | <.0001 |  | 1.579 | 0.3674 | <.0001 |
| **Study** | **Coulibaly 2017** | -0.02108 | 0.245 | 0.932 | **Coulibaly 2018** | 0.6602 | 0.416 | 0.113 |
|  | **Garba 2007** | 3.2555 | 0.2474 | <.0001 | **Garba 2007** | 0.3277 | 0.4107 | 0.425 |
|  | **Garba 2013** | 3.1392 | 0.2333 | <.0001 | **Garba 2009** | 1.1772 | 0.4111 | 0.004 |
|  | **Olliaro 2007** | 1.3427 | 0.2467 | <.0001 | **Garba 2013** | 0.3261 | 0.3853 | 0.397 |
|  | **Raso 2004** | -0.4346 | 0.2941 | 0.14 | **Olds, 1999** | 2.2346 | 0.426 | <.0001 |
|  | **Scherrer 2007** | -0.4288 | 0.2794 | 0.125 | **Lossa 1996** | 0.5047 | 0.3992 | 0.206 |
|  | **Sousa-Figueiredo 2012** | 3.3957 | 0.2119 | <.0001 | **Mutapi 2010** | 0.911 | 0.4118 | 0.027 |
|  | **Utzinger 1997** | -0.4721 | 0.2646 | 0.075 | **Niame 1995** | 0.8109 | 0.4346 | 0.062 |
|  | **Coulibaly 2011** | 0 | . | . | **Stete 2010** | 1.1438 | 0.4388 | 0.009 |
|  |  |  |  |  | **Coulibaly 2011** | 0 | . | . |
| **Age** | **[6-14]** | 0.3455 | 0.1207 | 0.004 | **[6-14]** | 0.5205 | 0.1691 | 0.002 |
|  | **[0-6[** | 0 | . | . | **[0-6[** | 0 | . | . |
| **Sex** | **Male** | -0.01176 | 0.07338 | 0.873 | **Male** | 0.1936 | 0.07726 | 0.012 |
|  | **Female** | 0 | . | . | **Female** | 0 | . | . |
