# Supplementary material for: Efficacy and safety of single-dose 40 mg/kg oral praziquantel in the treatment of schistosomiasis in preschool-age versus school-age children: An individual participant data meta-analysis
Source: PLoS Negl Trop Dis. 2020 Jun 22;14(6):e0008277. doi: 10.1371/journal.pntd.0008277 (PMC7360067; doi:10.1371/journal.pntd.0008277)
Supplement: S7 Table — Results of general linear model of individual S. mansoni ERR without baseline log transformed egg count as covariate and studies and age as factors (a: 3 categories, b: 2 categories). (DOCX) [file pntd.0008277.s007.docx]

S7 table. General linear model of individual ERR without baseline log transformed egg count as covariate and studies and age as factors (a: 3 categories, b: 2 categories)

| a |  | ***S. mansoni*** | | |  | ***S. haematobium*** | | |
| --- | --- | --- | --- | --- | --- | --- | --- | --- |
| **Effect** | **Category** | **Mean** | **SD** | **Pr > \|t\|** | **Category** | **Mean** | **SD** | **Pr > \|t\|** |
| **Intercept** |  | 0.9776 | 0.04696 | <.0001 |  | 0.9994 | 0.05608 | <.0001 |
| **STUDY** | **Coulibaly 2017** | -0.137 | 0.05848 | 0.0193 | **Coulibaly 2018** | -0.02778 | 0.06387 | 0.6637 |
|  | **Garba 2007** | -0.2456 | 0.05938 | <.0001 | **Garba 2007** | -0.00615 | 0.06303 | 0.9223 |
|  | **Garba 2013** | -0.1161 | 0.05552 | 0.0368 | **Garba 2009** | -0.03883 | 0.06312 | 0.5385 |
|  | **Olliaro 2007** | -0.06844 | 0.06327 | 0.2797 | **Garba 2013** | -0.03543 | 0.05914 | 0.5492 |
|  | **Raso 2004** | -0.2056 | 0.07106 | 0.0039 | **Olds, 1999** | -0.2629 | 0.06544 | <.0001 |
|  | **Scherrer 2007** | -0.1242 | 0.06695 | 0.0639 | **Lossa 1996** | -0.3312 | 0.06128 | <.0001 |
|  | **Sousa-Figueiredo 2012** | -0.1722 | 0.05038 | 0.0007 | **Mutapi 2010** | -0.06472 | 0.06324 | 0.3063 |
|  | **Utzinger 1997** | -0.1089 | 0.0646 | 0.0923 | **Niame 1995** | 0.03187 | 0.0668 | 0.6334 |
|  | **Coulibaly 2011** | 0 | . | . | **Stete 2010** | 0.01296 | 0.06751 | 0.8478 |
|  |  |  |  |  | **Coulibaly 2011** | 0 | . | . |
| **Age Categories** | **[10-14]** | 0.07165 | 0.03731 | 0.0551 | **[10-14]** | -0.03479 | 0.02722 | 0.2014 |
|  | **[6-10]** | 0.05147 | 0.02901 | 0.0762 | **[6-10]** | -0.02409 | 0.02648 | 0.3632 |
|  | **[0-6[** | 0 | . | . | **[0-6[** | 0 | . | . |

| b |  | ***S. mansoni*** | | |  | ***S. haematobium*** | | |
| --- | --- | --- | --- | --- | --- | --- | --- | --- |
| **Effect** | **Category** | **Mean** | **SD** | **Pr > \|t\|** | **Category** | **Mean** | **SD** | **Pr > \|t\|** |
| **Intercept** |  | 0.9776 | 0.04695 | <.0001 |  | 0.9994 | 0.05608 | <.0001 |
| **STUDY** | **Coulibaly 2017** | -0.1336 | 0.05827 | 0.0221 | **Coulibaly 2018** | -0.02743 | 0.06386 | 0.6676 |
|  | **Garba 2007** | -0.2396 | 0.05876 | <.0001 | **Garba 2007** | -0.00654 | 0.06303 | 0.9174 |
|  | **Garba 2013** | -0.1161 | 0.0555 | 0.0367 | **Garba 2009** | -0.0399 | 0.0631 | 0.5272 |
|  | **Olliaro 2007** | -0.05136 | 0.05859 | 0.3809 | **Garba 2013** | -0.03543 | 0.05913 | 0.5492 |
|  | **Raso 2004** | -0.1967 | 0.06995 | 0.005 | **Olds, 1999** | -0.2649 | 0.06539 | <.0001 |
|  | **Scherrer 2007** | -0.1183 | 0.06642 | 0.0753 | **Lossa 1996** | -0.3318 | 0.06127 | <.0001 |
|  | **Sousa-Figueiredo 2012** | -0.1731 | 0.05035 | 0.0006 | **Mutapi 2010** | -0.0632 | 0.06321 | 0.3175 |
|  | **Utzinger 1997** | -0.09834 | 0.06289 | 0.1182 | **Niame 1995** | 0.02891 | 0.06669 | 0.6647 |
|  | **Coulibaly 2011** | 0 | . | . | **Stete 2010** | 0.0093 | 0.06735 | 0.8902 |
|  |  |  |  |  | **Coulibaly 2011** | 0 | . | . |
| **Age Categories** | **[6-14]** | 0.05457 | 0.02867 | 0.0573 | **[6-14]** | -0.02826 | 0.02596 | 0.2764 |
|  | **[0-6[** | 0 | . | . | **[0-6[** | 0 | . | . |
