# Supplementary material for: Efficacy and safety of single-dose 40 mg/kg oral praziquantel in the treatment of schistosomiasis in preschool-age versus school-age children: An individual participant data meta-analysis
Source: PLoS Negl Trop Dis. 2020 Jun 22;14(6):e0008277. doi: 10.1371/journal.pntd.0008277 (PMC7360067; doi:10.1371/journal.pntd.0008277)
Supplement: S8 Table — Results of mixed model of individual S. haematobium ERR without baseline log transformed egg count as covariate and age as a fixed factor, with studies as a random factor (a: 3 categories, b: 2 categories). (DOCX) [file pntd.0008277.s008.docx]

S8 table. Mixed model of individual ERR without baseline log transformed egg count as covariate and age as a fixed factor, with studies as a random factor (a: 3 categories, b: 2 categories)

| a |  | ***S. mansoni*** | | |  | ***S. haematobium*** | | |
| --- | --- | --- | --- | --- | --- | --- | --- | --- |
| **Effect** | **Category** | **Mean** | **SD** | **Pr > \|t\|** | **Category** | **Mean** | **SD** | **Pr > \|t\|** |
| **Intercept** |  | 0.8505 | 0.029 | <.0001 |  | 0.9271 | 0.04336 | <.0001 |
| **Age Categories** | **[10-14]** | 0.06258 | 0.03243 | 0.0539 | **[10-14]** | -0.03681 | 0.02644 | 0.164 |
|  | **[6-10]** | 0.03855 | 0.02674 | 0.1498 | **[6-10]** | -0.02625 | 0.02575 | 0.3082 |
|  | **[0-6[** | 0 | . | . | **[0-6[** | 0 | . | . |

| b |  | ***S. mansoni*** | | |  | ***S. haematobium*** | | |
| --- | --- | --- | --- | --- | --- | --- | --- | --- |
| **Effect** | **Category** | **Mean** | **SD** | **Pr > \|t\|** | **Category** | **Mean** | **SD** | **Pr > \|t\|** |
| **Intercept** |  | 0.8556 | 0.02898 | <.0001 |  | 0.9263 | 0.04332 | <.0001 |
| **Age Categories** | **[6-14]** | 0.04525 | 0.02589 | 0.0808 | **[6-14]** | -0.03048 | 0.02518 | 0.2264 |
|  | **[0-6[** | 0 | . | . | **[0-6[** | 0 | . | . |
