# Supplementary material for: Efficacy and safety of single-dose 40 mg/kg oral praziquantel in the treatment of schistosomiasis in preschool-age versus school-age children: An individual participant data meta-analysis
Source: PLoS Negl Trop Dis. 2020 Jun 22;14(6):e0008277. doi: 10.1371/journal.pntd.0008277 (PMC7360067; doi:10.1371/journal.pntd.0008277)
Supplement: S10 Table — (DOCX) [file pntd.0008277.s010.docx]

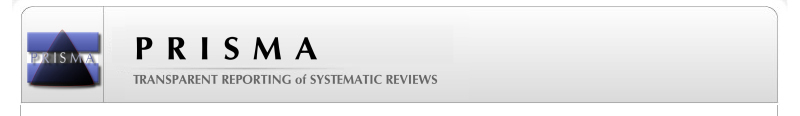
**PRISMA 2009 Flow Diagram**

Studies included in quantitative synthesis (meta-analysis)
(n =16)

Studies included in qualitative synthesis
(n =16)

Full-text articles excluded, with reasons
(n =7)

Full-text articles assessed for eligibility
(n =23)

Records excluded
(n =77)

Records screened
(n =90)

Records after duplicates removed
(n =90)

Additional records identified through other sources
(n =0)

## Identification

## Eligibility

## Included

## Screening

Records identified through database searching
(n =90)
