# Supplementary figures and images for: Efficacy and safety of single-dose 40 mg/kg oral praziquantel in the treatment of schistosomiasis in preschool-age versus school-age children: An individual participant data meta-analysis
Source: PLoS Negl Trop Dis. 2020 Jun 22;14(6):e0008277. doi: 10.1371/journal.pntd.0008277 (PMC7360067; doi:10.1371/journal.pntd.0008277)

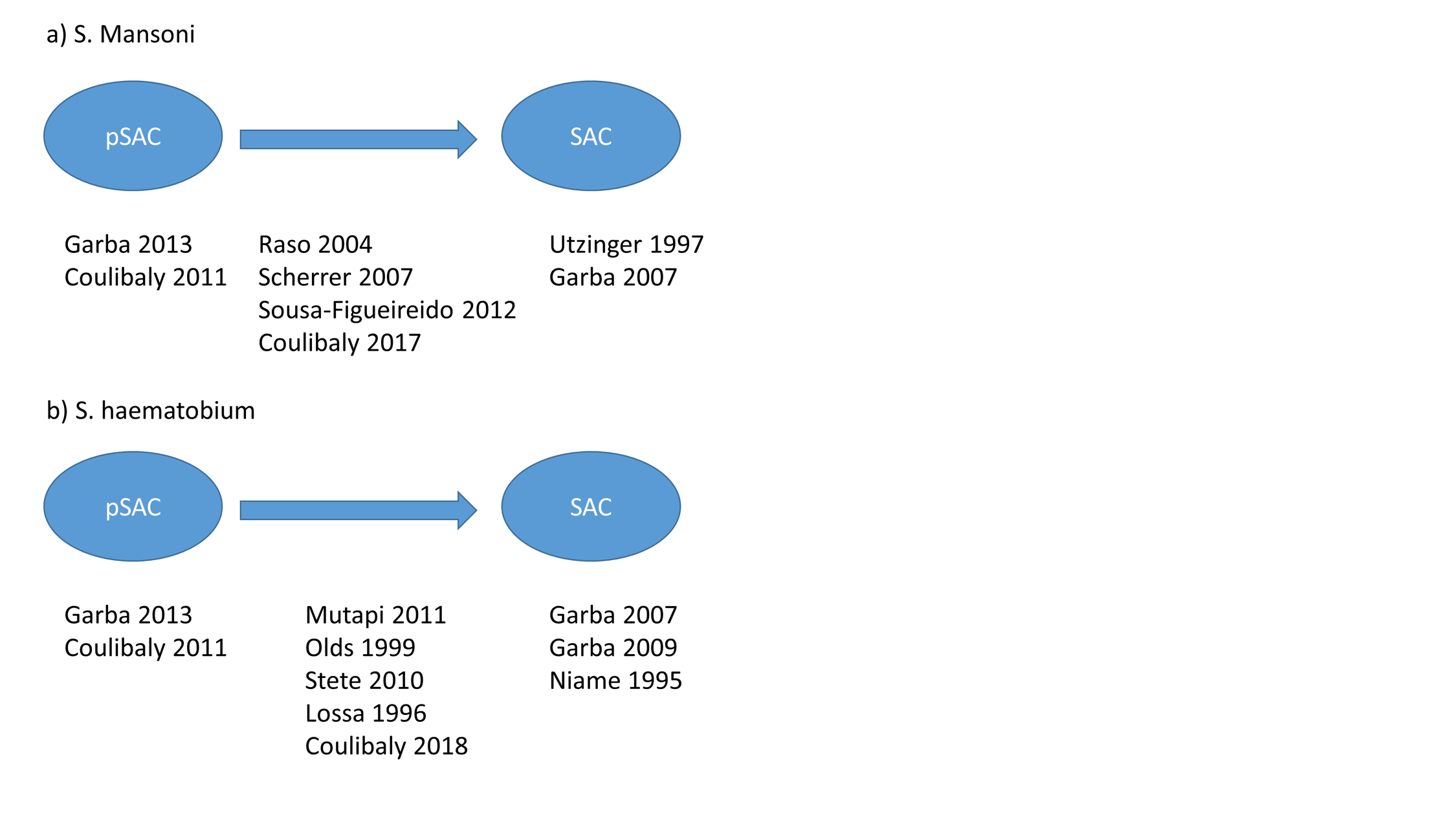

Supplement: S1 Fig — (TIF) [file pntd.0008277.s011.tif]

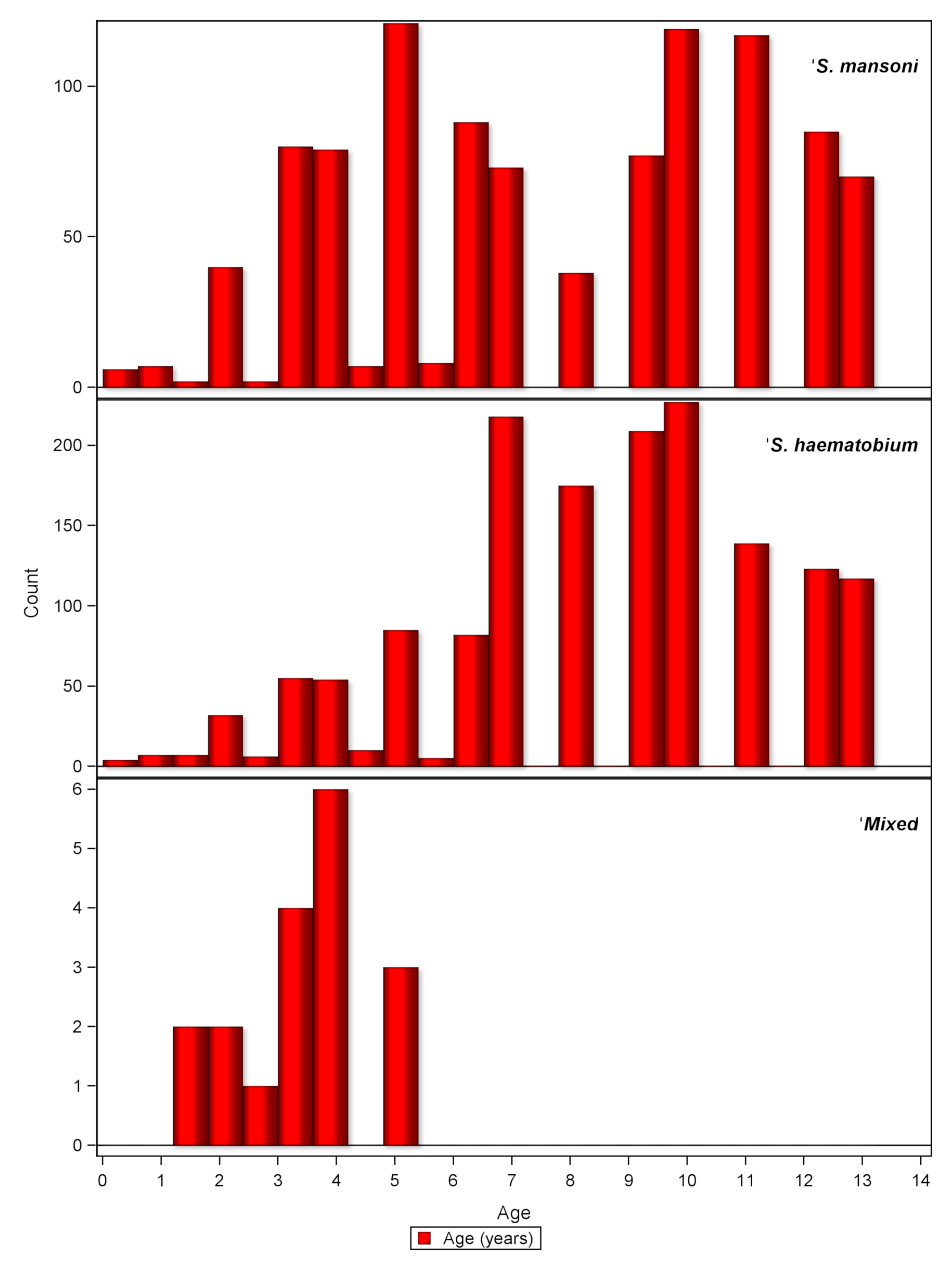

Supplement: S2 Fig — (TIF) [file pntd.0008277.s012.tif]
