# Supplementary material for: Efficacy and safety of single-dose 40 mg/kg oral praziquantel in the treatment of schistosomiasis in preschool-age versus school-age children: An individual participant data meta-analysis
Source: PLoS Negl Trop Dis. 2020 Jun 22;14(6):e0008277. doi: 10.1371/journal.pntd.0008277 (PMC7360067; doi:10.1371/journal.pntd.0008277)
Supplement: Attachment — Submitted filename: PNTD-D-19-02157_Response v05.docx [file pntd.0008277.s013.docx]

**Olliaro PL *et al.* “Efficacy and safety of single 40 mg/kg oral praziquantel in the treatment of schistosomiasis in preschool-age versus school-age children: an individual participant data meta-analysis” (your manuscript no. PNTD-D-19-02157**

Oxford and Luxembourg, 3^rd^ April 2020

Dear Drs Montresor and Geary

We were delighted to receive your e-mail dated 5 March 2020 and thank you and the three external reviewers for the careful examination of the aforementioned manuscript. In the meantime, we have revised our manuscript in the light of the reviewers’ suggestions.

Below, please find our point-by-point response, clearly indicating how and where in the manuscript changes have been made. As requested, we enclose two versions of our revised manuscript: (i) a track-changed version and (ii) a final clean version. Please note that the line numbers indicated below refer to the final clean version.

We look forward to your further disposition.

Yours sincerely,

Piero L. Olliaro & Michel Vaillant

(on behalf of all authors)

************************************************************************************

**Reviewer's Responses to Questions**

***Methods:***

-Are the objectives of the study clearly articulated with a clear testable hypothesis stated?

-Is the study design appropriate to address the stated objectives?

-Is the population clearly described and appropriate for the hypothesis being tested?

-Is the sample size sufficient to ensure adequate power to address the hypothesis being tested?

-Were correct statistical analysis used to support conclusions?

-Are there concerns about ethical or regulatory requirements being met?

**Reviewer #1:** Did all studies deliver praziquantel to the PSACs in the same way - whole tablet, crushed tablet in juice?

Response: yes, in general crushed tablets

Were all treatments accompanied by food ? If not, did this have an impact on adverse events?

Response: yes, in general with food

Was the praziquantel used in the studies all from the same source / manufacturer?

Response: product origins varied

I see little value including S. japonicum due to the stated absence of data.

Response: Agreed. Deleted

**Reviewer #2:** A very clear statistical analysis from a well-organised database of IPD.

Response: We thank the reviewer for this much appreciated comment on our work.

**Reviewer #3**: General

1) Were PRISMA guidelines used for this Meta-analysis. Not referenced in methods. You provide the flow diagram but should reference in methods.

Response: the PRISMA guidelines were used and followed for reporting the current work. Added in the manuscript.

2) Regarding S. japonicum, there is only ONE PSAC subjects across all studies. One PSAC in China, none in Philippines. How is this useful for meta with focus on treatment safety and efficacy in PSAC??? At the very least references to S. japonicum should be removed from abstract and other places.

Response: Agreed: deleted

3) in many tables the close brackets [ ] is left open [ [ for many age groups

Response: Yes because the last number is not included in the interval. This is the typical significance of brackets.

5) In methods, please clarify what was asked regarding AEs at follow up. Did you ask about current AE due to the (weeks preceding) treatment or recall of AE in day or so after?

Response: It is difficult to generalize as this varied across centres. For instance, in Zimbabwe-all treated children were asked to remain at the treatment centre for 1 hour after administering the medication to assess if the PZQ treatment had immediate side effects and to determine whether the medication was lost through vomiting by the children. In addition, Parents/guardians reported back 24 hours after PZQ treatment (preferably with the child) to answer a questionnaire on side effects following PZQ administration

6) Methods do not approach whether studies referenced repeating dose if vomiting occurred or rates of refusal among young children. Although some studies likely didn’t collect, one can still add to discussion regarding ways to improve overall ERR for all children.

Response: reportedly vomiting or refusal a rare event in these studies. However, situation in programmes is likely different.

See point 6 above. Lines 153-55. It is not clear here whether you are referring to reporting bias (participant attribute) or publication bias. These lines seem to apply to the latter.

Response: The assessment of methodological quality refers to the quality of the methodological design and reporting of it in each study - not reporting bias or publication bias only.

Specific

Line 164-spell out AM-ERR with first use

Response: Arithmetic Mean – Egg Reduction Rate. This was changed in the text.

Line 233- as worded it is not clear how you are supporting lack of different types of bias:

“The RCTs were at low risk of selection bias with computer-generated block randomization, adequate allocation concealment, and blinding of either participants, personnel, or outcome assessment.”

Some of these procedures do not support “selection bias”

Response: Heterogeneity was accounted for in the models by using random effects. See statistical methods.

--------------------

***Results:***

-Does the analysis presented match the analysis plan?

-Are the results clearly and completely presented?

-Are the figures (Tables, Images) of sufficient quality for clarity?

**Reviewer #1**: OK

**Reviewer #2**: Some excellent graphs showing novelty in visualization of complex data.

**Reviewer #3**: See general comments on S. japonicum Lines 310-12 This sentence is not clear:

At least one AE was experienced at 4 hours post-treatment by 19% and 44% of under 6-year-old children and children aged 10-14 years, respectively.

Response: Modified to read: “At 4 hours post-treatment, 19% of under 6-year-old children and 44% of children aged 10-14 were reported to have experienced at least one AE.”

Table 6

Please provide clearer title and column headings to denote what the “mean” represents

Response: Modified in table 6a and 6b.

Figure 6 – need to clarify what different colors represent in the bar graph. Large number of low ERR in one study? As above, this also needs to be discussed. Neither the figure legend nor results text explain this figure at all adequately.

Response: Color coding in the legend for each study. The same colors are used for the bar graphs and the scatterplots. Keys of interpretation are now inserted in the text just after fig. 5

In many tables the close brackets [ ] is left open [ [ for many age groups

Response: Yes because the last number is not included in the interval. This is typical significance of brackets.

--------------------

***Conclusions:***

-Are the conclusions supported by the data presented?

-Are the limitations of analysis clearly described?

-Do the authors discuss how these data can be helpful to advance our understanding of the topic under study?

-Is public health relevance addressed?

**Reviewer #1**: OK

**Reviewer #2**: Large true but I think the authors should elaborate a bit more on the following:

Egg count is not the 'best' measure of cure in young children (since there is an unusual dynamic across time of first infection and age towards patency (see <https://www.ncbi.nlm.nih.gov/pubmed/21245910>). I realize that egg-count is important in SAC dynamics but some awareness of this non-linear effect in PSAC is important.

Response: The following sentence has been added: “A more general methodological issue is the limitations of counting eggs in excreta in young children, and the imperfect correlation between egg shedding and worm burden, which would be better reflected by antigen-detection methods. [Ref: Stothard et al, 2011]”

By that same token a bit more should be made of antigen biomarkers (either CAA in sera/urine or CCA in urine).

Response: The following sentence has been added: “However, the significance and comparability of results of efficacy studies based on antigen-detection methods vis-à-vis direct egg-detection remains unclear.”

Collectively I expect the use of biomarkers would show less efficacy, and note that the paediatric praziquantel consortium (on the basis of their trials) is adopting 50 mg/kg of the L-PZQ (given inferiority/superiority measures).

The future adoption of a mono-isomeric form in PSAC somewhat changes the present guidance that this current script offers. It does support however the WHO-stop gap of using crushed racemate in this age class. From the results presented here seems to be a sensible altrnative way forward more immediate to the L-PZQ (which might need testing in SAC?).

**Reviewer #3**: Lines 340-1- same issue on emphasis on formulation. Most of your included studies used crushed tablets

Lines 365-

Discussion focuses too heavily on lack of difference in other studies at 60 mg/kg. Though you show that the ERR not significantly different between age groups at 40 mg/kg, there is not discussion of the fact that most studies did not find ERR at or above WHO recommended 95% threshold. This must be discussed. There are other reasons besides dose provided that may explain including different dosing strategies (larger total dose over more time to improve tolerability, formulations that might improve drug delivery based on tolerability, absorption etc.

Response: The following sentence has been added: “It should also be noted that the WHO-recommended 95% ERR threshold was not met in man studies, which calls for improved dosing and delivery strategies.”

Also, at some point in the discussion, particularly when you reference issue of higher doses to 60 mg/kg, must state that much less is known about this in S. japonicum.

Response: Added as limitation in the Discussion

Here or elsewhere, must discuss the almost double rate of ERR less than 0% in S. mansoni among PSAC.

This can be included in above regarding adequacy of ANY current does.

Response: Added in the Discussion “This in particular considering preschool-age children have about twice as high rates of non-responders in the individual-patient ERR analysis than older children.”

Lines379+ - must include in limitations that you cannot say anything about comparing efficacy of 40 mg/kg among PSAC and school age for S. japonicum. That gets lost entirely.

Response: Added as limitation in the Discussion

--------------------

***Editorial and Data Presentation Modifications?***

Use this section for editorial suggestions as well as relatively minor modifications of existing data that would enhance clarity. If the only modifications needed are minor and/or editorial, you may wish to recommend “Minor Revision” or “Accept”.

**Reviewer #1**: Apart from the comments above "Accept"

**Reviewer #2**: NA

**Reviewer #3**: Methods

Already responded above

--------------------

***Summary and General Comments:***

Use this section to provide overall comments, discuss strengths/weaknesses of the study, novelty, significance, general execution and scholarship. You may also include additional comments for the author, including concerns about dual publication, research ethics, or publication ethics. If requesting major revision, please articulate the new experiments that are needed.

**Reviewer #1**: The limitations of the study have been addressed by the authors. This is a most useful analysis of data considered in previously published papers and highlights that treatment of PSACs with praziquantel has a similar benefits to treatment of older children.

**Reviewer #2**: A useful script - secondary analysis - competently done. For brevity I think it could be shortened a little will a few more specific pointers on 'grey' areas (mono-isomer v racemate) where future impact might be less than expected.

**Reviewer #3**: 1) Were PRISMA guidelines used for this Meta-analysis. Not referenced in methods. You provide the flow diagram but should reference in methods.

Response: already addressed above

2) Regarding S. japonicum, there is only ONE PSAC subjects across all studies. One PSAC in China, none in Philippines. How is this useful for meta with focus on treatment safety and efficacy in PSAC??? At the very least references to S. japonicum should be removed from abstract and other places.

Response: already addressed above

3) introduction Lines 97-101: Same issue on waiting for other formulation. You cite the WHO report of 2010 on this which actually states in the executive summary:

“Studies on the treatment of preschool-age children conducted in these five countries (n=3198) among children aged 1 month to 7 years showed that praziquantel in a tablet or suspension formulation was safe and effective against schistosomiasis, and acceptable…The two studies in Mali and Uganda that compared suspension and tablets found no difference in cure rates between the two formulations. In Uganda, there was also no difference in rates of egg reduction between the two formulations.”

This is not simply an issue of semantics; the way this manuscript reads strongly implies that we must await pediatric formulations which will do harm to young children who can take crushed tablets. Even your PICOS statement addresses efficacy of 40 mg/kg dose as the primary goal of the study, without reference to dosing form which dominates abstract and introduction.

Response: last sentence of the Abstract modified to read “Preventive chemotherapy might therefore be extended to preschool-age children, with proper monitoring of its efficacy and safety.”

Sentence modified accordingly in the plain-language summary

We also modified the sentence in the Introduction to read “The reason is that the current formulation (large, bitter tasting 600 mg tablets), although often crushed and dissolved in practice, is unsuited for use in young children, and hence, efforts are underway to develop an orally dispersible tablet formulation for young children.”

4) Introduction Lines 99-101- at least one drug (Bayer) is “registered” (US FDA approved) down to age one so this should be modified accordingly.

Response: sentence deleted for simplicity.

4) In methods, please clarify what was asked regarding AEs at follow up. Did you ask about current AE due to the (weeks preceding) treatment or recall of AE in day or so after?

Response: already addressed above

5) at some point in the discussion, particularly when you reference issue of higher doses to 60 mg/kg, must state that much less is known about this in S. japonicum.

Response: already addressed above

6) Discussion focuses too heavily on lack of difference in other studies at 60 mg/kg. Though you show that the ERR not significantly different between age groups at 40 mg/kg, there is not discussion of the fact that most studies did not find ERR at or above WHO recommended 95% threshold. This must be discussed. There are other reasons besides dose provided that may explain including different dosing strategies (larger total dose over more time to improve tolerability, formulations that might improve drug delivery based on tolerability, absorption etc.

Response: already addressed above

7) if any authors receive funding from pharmaceutical companies developing Pediatric formulations for Praziquantel, this should be disclosed.

Response: none declared

8) for S. mansoni, there were double the number of children who had ERR 0%. This is never discussed and provides good topic for discussion under tolerability issues in this age group. Did they vomit the dose/refuse?

Response: already addressed above

v

9) Methods do not approach whether studies referenced repeating dose if vomiting occurred or rates of refusal among young children. Although some studies likely didn’t collect, one can still add to discussion regarding ways to improve overall ERR for all children. See point 6 above.

Response: already addressed above
